# Effective playing time affects physical match performance in soccer: An analysis according to playing position

**DOI:** 10.5114/biolsport.2023.123320

**Published:** 2023-02-03

**Authors:** Stefan Altmann, Leon Forcher, Alexander Woll, Sascha Härtel

**Affiliations:** 1 Institute of Sports and Sports Science, Karlsruhe Institute of Technology, Karlsruhe, Germany; 2 TSG ResearchLab gGmbH, Zuzenhausen, Germany; 3 TSG 1899 Hoffenheim, Zuzenhausen, Germany

**Keywords:** Football, Physical match performance, Ball in play, Fatigue, Sprint, Acceleration, High intensity, Position

## Abstract

This study aimed to analyse the influence of effective playing time on physical match performance according to playing position in professional soccer. Official match data from 267 matches (3,731 single observations) during the 2018/2019 season of the German Bundesliga were used and the effective playing time (duration of play after subtracting the time taken up by stoppages, substitutions, injuries, and goals) was captured for each match. The physical match performance parameters total distance, high-intensity distance, sprinting distance, maximum velocity, and accelerations were analysed. Players were categorized as central defender, wide defender, central defensive midfielder, central offensive midfielder, wide midfielder, and forward. Effective playing time influenced physical match performance, with total distance and accelerations (r = 0.48–0.61) being the most and high-intensity distance, sprinting distance, and maximum velocity (r = -0.17–0.03) the least affected parameters. Players covered on average 10% more total distance and performed 13% more accelerations, while sprinting 7–10% less in matches with long (> 65 min) compared to short (< 50 min) effective playing times. The influence of effective playing time was rather similar between playing positions. Still, physical performance of wide midfielders and forwards partly deviated from the pattern observed in the other positions. Coaches and practitioners should be aware that effective playing time influences physical match performance in the German Bundesliga, while special attention should be given to wide midfielders and forwards. Effective playing time and its general and position-specific effects should be taken into account when interpreting physical match performance, thereby facilitating load management practices and training design.

## INTRODUCTION

The physical match performance of professional soccer players based on parameters such as running at different velocities, sprinting, accelerating, and decelerating has been investigated in a plethora of studies [1, 2]. While an official match lasts for 90 min plus additional time, several interruptions due to substitutions, fouls, injuries, goals, or the ball being out of play commonly occur over the course of a match [3, 4]. Taking these interruptions into account, the net playing time or effective playing time decreases to 52–55 min [5–7]. In this regard, studies have shown that physical match performance (e.g., running distances in different velocity zones per minute) is higher during the effective playing time compared to when the ball is out of play or to total playing time [8–10]. Moreover, considering effective playing time also sheds some light on the typically observed reduction in physical performance during the second half of a match, which has traditionally been linked to fatigue [11]. Here, studies revealed that lower performance in the second half could be explained in large part by the number and length of game interruptions [4] or even that performance was maintained when accounting for these interruptions or effective playing time [7, 12]. However, these results differ according to playing position; e.g., in contrast to other playing positions, forwards seem to reduce their match performance in the second half despite reductions in effective playing being considered [7].

While effective playing time accounts for 62% of total playing time on average, it might range between 49% and 72%, thereby demonstrating considerable variability [3]. Therefore, it seems plausible that physical match performance might differ according to effective playing time. Nevertheless, to date, there is no study investigating this potential association.

Therefore, the aim of this short communication was to analyse the influence of effective playing time on physical match performance in soccer according to playing position based on a full season in a European top league. We hypothesized that physical match performance would be influenced by effective playing time [4, 12] and that there emerged positional differences in the way players adapt to alterations in effective playing time [7, 13]. The results of this study could facilitate the interpretation of physical match performance in relation to effective playing time, and consequently improve load management practices and training design in professional soccer.

## MATERIALS AND METHODS

### Study Design

In the present study, official match data from 267 matches during the 2018/2019 season of the German Bundesliga were used to determine the influence of effective playing time on physical match performance according to playing position.

### Subjects

A total of 267 out of 306 matches were analysed, as matches including a player dismissal were not considered. Only outfield players who participated in the whole game time (i.e., the full 90 min) of the respective match were included. This led to a maximum of 20 outfield players per match and a total of 474 players and 3,731 separate observations (i.e., a single match performance of one player) that were analysed. All players were distinguished into six different playing positions as follows [14, 15]: central defender (1,130 observations), wide defender (833 observations), central defensive midfielder (563 observations), central offensive midfielder (390 observations), wide midfielder (400 observations), forward (415 observations).

The study was conducted according to the guidelines of the Declaration of Helsinki and was approved by the local ethics committee.

### Procedures

All data used in this study are based on match analysis data of the German Bundesliga (DFL Observed Tracking-Data, Bundesliga 2018/2019, Deltatre, Turin, Italy). The data were captured using a Multi-Camera-Tracking System (TRACAB, Chyron Hego, Melville, NY, USA), which has recently been validated for obtaining game analysis data [16]. To assess the physical performance of the players, the parameters total distance [km], high-intensity distance [km], sprinting distance [km], maximum velocity [km/h], and number of accelerations [quantity] were analysed. As with previous studies [17–19], high-intensity distance was defined as 17.00–23.99 km/h and sprinting distance as ≥ 24.00 km/h. The maximum velocity reflects the highest velocity reached throughout a game. One acceleration was counted, when there were positive acceleration values in each frame for ≥ 1.5 s. All definitions are based on the catalogue of the German soccer league [20]. Lastly, the effective playing time of each match was captured, defined as the duration of play after subtracting the time taken up by stoppages, substitutions, injuries, and goals [7, 12].

### Statistical Analysis

The data were analysed using SPSS statistical software version 28.0 (IBM SPSS, IBM Corporation, Armonk, NY). Mean values and standard deviations (SD) for physical match performance were calculated at the team level and for each playing position separately.

Pearson’s product-moment correlations (r) and 95% confidence intervals were used to determine the association between effective playing time and physical match performance. According to Hopkins [21], the magnitude of the correlation coefficient was considered as trivial (r < 0.1), small (0.1 ≤ r < 0.3), moderate (0.3 ≤ r < 0.5), large (0.5 ≤ r < 0.7), very large (0.7 ≤ r < 0.9), and nearly perfect (r ≥ 0.9). Lastly, possible differences in physical match performance between effective playing time categories (< 50 min, 50–55 min, 56–60 min, 61–65 min, > 65 min) were analysed using one-way analysis of variance and subsequent Bonferroni post-hoc comparisons.

For the applied statistical procedures, the assumption of a normal distribution was made. The significance level for all statistical tests was set a priori to 0.05.

## RESULTS

Descriptive values for each physical match performance parameter according to playing position can be found in Table 1. Effective playing time was 57.45 ± 4.31 min, with a minimum of 47.00 min and a maximum of 70.58 min.

**TABLE 1:** Descriptive data for the physical match performance parameters total distance, high-intensity distance, sprinting distance, maximum velocity, and number of accelerations for the total sample and separated by playing position. Results are presented as mean values ± SD.

	Total distance [km]	High-intensity distance [km]	Sprinting distance [km]	Maximum velocity [km/h]	Number of accelerations
Total sample (n = 3,731)	10.94 ± 0.88	1.35 ± 0.37	0.28 ± 0.14	31.02 ± 1.63	495 ± 41
CD (n = 1,130)	10.24 ± 0.64	1.00 ± 0.22	0.19 ± 0.09	30.71 ± 1.68	480 ± 36
WD (n = 833)	10.90 ± 0.66	1.42 ± 0.27	0.36 ± 0.13	31.56 ± 1.47	503 ± 37
CDM (n = 563)	11.60 ± 0.68	1.49 ± 0.33	0.22 ± 0.10	30.22 ± 1.58	518 ± 38
COM (n = 390)	11.72 ± 0.70	1.67 ± 0.29	0.32 ± 0.13	30.92 ± 1.52	507 ± 42
WM (n = 400)	11.34 ± 0.74	1.61 ± 0.28	0.40 ± 0.15	31.72 ± 1.43	499 ± 39
FW (n = 415)	10.86 ± 0.83	1.44 ± 0.32	0.34 ± 0.12	31.28 ± 1.47	469 ± 41

SD – Standard deviation; n = Number of single observations; CD – Central defender; WD – Wide defender; CDM – Central defensive midfielder; COM – Central offensive midfielder; WM – Wide midfielder; FW – Forward.

**FIG. 1:**
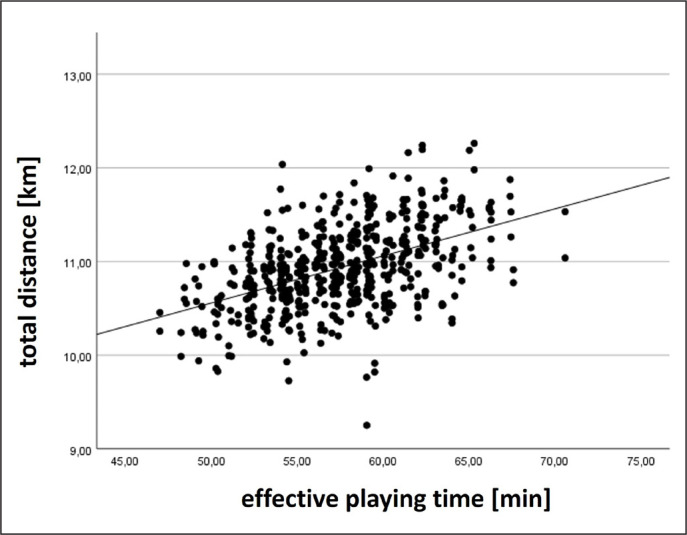
Relationship between total distance at the team level and effective playing time.

**FIG. 2:**
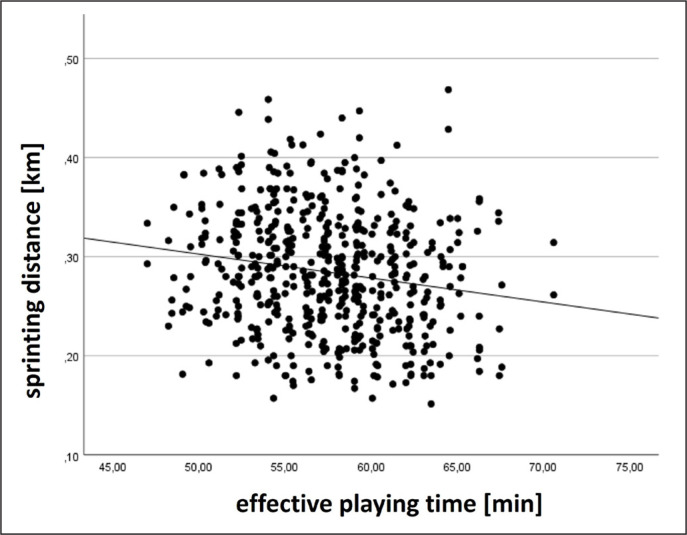
Relationship between sprinting distance at the team level and effective playing time.

At the team level, moderate to large positive relationships with effective playing time were found for total distance and accelerations, trivial negative relationships for sprinting distance and maximum velocity, and a trivial positive relationship for high-intensity distance. Exemplarily, results for total distance and sprinting distance at the team level are displayed in Figures 1–2.

Wide midfielders demonstrated the smallest positive relationships for total distance and accelerations, as well as the smallest negative relationships for maximum velocity and alongside central offensive midfielders for sprinting distance as compared to the other playing positions. By contrast, forwards were the playing position that showed the largest negative relationships for sprinting distance and maximum velocity (Table 2).

**TABLE 2:** Pearson’s r, 95% CI and p-values for correlations between the effective playing time and the physical match performance parameters total distance, high-intensity distance, sprinting distance, maximum velocity, and number of accelerations on a team level and separated by playing position.

		Total distance	High-intensity distance	Sprinting distance	Maximum velocity	Number of accelerations
Whole team (n* = 267)	Pearson’s r	0.48	0.03	-0.17	-0.13	0.61
	95% CI	0.41–0.55	-0.06–0.11	-0.25– -0.09	-0.21– -0.04	0.55–0.66
	p-value	< 0.01	0.51	< 0.01	< 0.01	< 0.01

CD (n = 1,130)	Pearson’s r	0.35	-0.02	-0.12	-0.05	0.54
	95% CI	0.30–0.40	-0.08–0.3	-0.17– -0.62	-0.11–0.01	0.49–0.57
	p-value	< 0.01	0.45	< 0.01	0.09	< 0.01

WD (n = 833)	Pearson’s r	0.32	-0.03	-0.12	-0.06	0.49
	95% CI	0.26–0.38	-0.10–0.04	-0.20– -0.05	-0.13–0.02	0.44–0.53
	p-value	< 0.01	0.45	< 0.01	0.10	< 0.01

CDM (n = 563)	Pearson’s r	0.39	-0.02	-0.10	-0.09	0.35
	95% CI	0.32–0.46	-0.11–0.06	-0.18– -0.02	-0.17– -0.01	0.28–0.42
	p-value	< 0.01	0.59	0.02	0.03	< 0.01

COM (n = 390)	Pearson’s r	0.34	0.05	-0.03	-0.02	0.42
	95% CI	0.25–0.43	-0.05–0.15	-0.13–0.07	-0.12–0.08	0.33–0.49
	p-value	< 0.01	0.33	0.61	0.66	< 0.01

WM (n = 400)	Pearson’s r	0.20	0.01	-0.04	< 0.01	0.34
	95% CI	0.11–0.30	-0.09–0.11	-0.14–0.06	-0.10–0.10	0.25–0.42
	p-value	< 0.01	0.88	0.43	0.99	< 0.01

FW (n = 415)	Pearson’s r	0.31	0.09	-0.21	-0.15	0.48
	95% CI	0.22–0.40	-0.01–0.18	-0.30– -0.12	-0.24– -0.05	0.40–0.55
	p-value	< 0.01	0.08	< 0.01	< 0.01	< 0.01

95% CI – 95% Confidence interval; n* = Number of matches; n = Number of single observations; CD – Central defender; WD – Wide defender; CDM – Central defensive midfielder; COM – Central offensive midfielder; WM – Wide midfielder; FW – Forward.

**FIG. 3:**
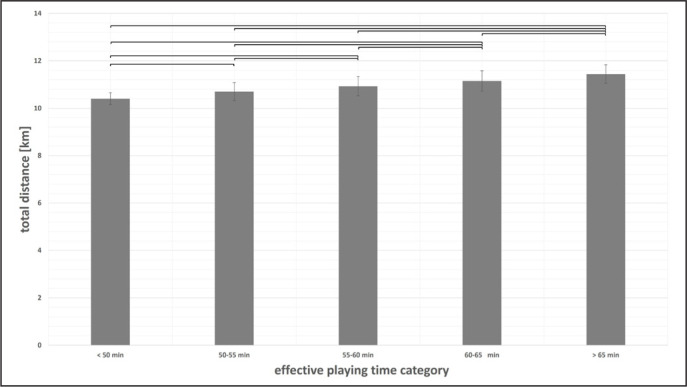
Total distance at the team level according to effective playing time category presented as mean values ± SD. Braces indicate significant differences between effective playing time categories.

**FIG. 4:**
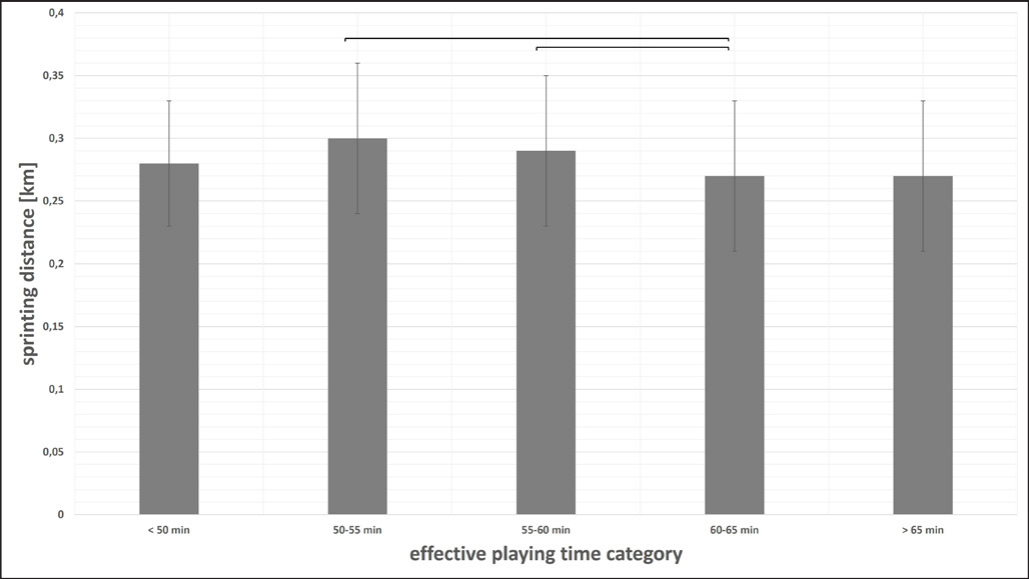
Sprinting distance at the team level according to effective playing time category presented as mean values ± SD. Braces indicate significant differences between effective playing time categories.

Differences in physical match performance between effective playing time categories were found for all parameters (ANOVA p < 0.01) except high-intensity distance (ANOVA p = 0.43). Exemplarily, results for total distance and sprinting distance at the team level are displayed in Figures 3–4. Cohen’s d effect sizes ranged between 0.52 and 3.18 for total distance, and between 0.00 and 0.50 for sprinting distance.

## DISCUSSION

The aim of this short communication was to analyse the influence of effective playing time on physical match performance according to playing position in professional soccer.

Our main finding was that effective playing time influenced physical match performance, with total distance and accelerations being the most and high-intensity distance, sprinting distance, and maximum velocity the least affected parameters. Therefore, our first hypothesis addressing the general influence of effective playing time can be confirmed. At the positional level, the influence of effective playing time was rather similar between playing positions. Nevertheless, physical match performance of wide midfielders and forwards partly deviated from the pattern observed in the other positions. While wide midfielders typically adjust their total distance and number of accelerations less in order to maintain their sprinting distance and maximum velocity when effective playing time is increased, forwards display the largest losses in sprinting distance and maximum velocity during such scenarios. Hence, our second hypothesis postulating differences between playing positions can only be partially confirmed.

Our findings on physical match performance according to playing position generally support previous studies from other professional European leagues [13, 17, 22–25]. For instance, both central defensive and offensive midfielders as well as wide midfielders covered the greatest total and high-intensity distance [13, 17, 25]. In addition, wide midfielders followed by wide defenders and forwards achieved the greatest sprinting distance, while central defensive midfielders and central defenders covered the shortest distances while sprinting [13, 17, 22, 23]. Deviations in the absolute distances between the present and previous studies might be explained by different definitions of parameters such as high-speed running and sprinting.

The effective playing time during the 2018/2019 season in the German Bundesliga was 57.45 ± 4.31 min on average, which is somewhat longer than what was reported for the Spanish La Liga 2018/2019 (52.3 ± 4.7 min) [7], Italian Serie A 2018/2019 (54 ± 11 min) [6], UEFA EURO 2008 (54.4 ± 4 minutes) [12], and FIFA World Cup 2014 (55.12 ± 5.37 min) [26]. Moreover, effective playing time showed high levels of variability, ranging from a minimum of 47.00 min to a maximum of 70.58 min. This result is well in line with previous evidence from Siegle et al. [3], who reported a range of 49% to 72% of total playing time in the 2009/2010 German Bundesliga season.

Our results further revealed that total distance and number of accelerations increased with increasing effective playing time independently of the playing position. Typically, outfield players cover around 1 km (10%) more total distance and perform 66 (13%) more accelerations in matches with long (> 65 min) compared to short (< 50 min) effective playing times. By contrast, actions executed at higher velocities seem to be less influenced by effective playing time. While there was only a non-significant trivial positive relationship regarding high-intensity distance, statistically significant trivial negative relationships were revealed for sprinting distance and maximum velocity. For example, sprinting distance was on average decreased by 20–30 m (7–10%) during matches with the highest effective playing time. In summary, while players increase their total distance and number of accelerations during matches with a longer effective playing time, high-intensity parameters remain rather stable or even slightly decrease.

One possible explanation for the latter finding might be that matches with shorter effective playing time are characterized by many breaks, allowing the players to recover and subsequently perform more sprints compared to matches with longer effective playing time and fewer breaks to recover. However, without additional data about the match context and the physical capabilities of the players, it remains unclear if this might be due to the players not being able to perform more actions of high intensity or if the typical match context when effective playing time is increased does not pressure them to do so.

When looking at each playing position separately, a rather similar pattern as compared to the one at the team level was evident. Nevertheless, wide midfielders somewhat deviated from this pattern. More specifically, they typically increased their total distance and number of accelerations by a smaller extent with increasing effective playing time, while best maintaining their sprinting distance and maximal velocity output compared to other playing positions. This observation could be interpreted as a pacing strategy of wide midfielders. In matches with short effective playing time, there are many game interruptions that allow the players to recover from preceding high-intensity actions. By contrast, longer effective playing times lead to fewer recovery opportunities, and this might affect playing positions differently. The mentioned wide midfielders are commonly subject to the highest physical match demands of all playing positions, as evidenced by the present results as well as previous studies [7, 13, 17, 25]. As such players do not necessarily possess better physiological capacities (e.g., aerobic endurance) compared to other positions, they might already experience a heavy internal load during matches with conventional effective playing times [27]. During matches with long effective playing time, they therefore might increase total distance and number of accelerations to only small extents in order to save resources for maintaining sprinting distance and maximum velocity, which are of utmost importance for their tactical duties [28]. Such a pacing strategy could not be observed for other positions which mainly showed larger increases in total distance and number of accelerations, while slightly decreasing sprinting distance and maximum velocity. In addition, forwards displayed the largest decreases of all playing positions for sprinting distance and maximum velocity when effective playing time was increased. However, as forwards commonly possess a rather balanced profile in terms of physical match demands [17, 25], more contextual information is needed to explain this finding. Indeed, as the observed differences between playing positions were rather small, these findings should not be overinterpreted.

The main strengths of this investigation are that the analysis was carried out for all teams over a whole season in a top European soccer league and that different playing positions were distinguished, leading to more contextualized results as compared to team analyses only. Moreover, while matches with red cards and players who were not involved in the whole match were excluded from the analysis, thereby leading to high quality of the data in this regard, there are some limitations of this study that should be acknowledged. First, we only looked at the entire match as opposed to distinguishing between playing halves or shorter periods of play (e.g., 15-min sections) [4, 7, 12]. Second, neither contextual factors like venue, opposition quality, or scoreline [5, 29, 30] nor the fitness level of the players were considered [31]. Addressing both aspects seems promising for future studies in order to gain a deeper understanding of the time course of the players’ adaptations to altered effective playing times as well as to contextual factors leading to such adaptations.

## CONCLUSIONS

Coaches and practitioners should be aware that effective playing time influences physical match performance in the German Bundesliga, with total distance and accelerations being increased, and high-intensity distance, sprinting distance, and maximum velocity being maintained or slightly decreased during matches with long effective playing time. Special attention should be given to wide midfielders, who typically adjust their total distance and number of accelerations less in order to maintain their sprinting distance and maximum velocity when effective playing time is increased, as well as to forwards, who display the largest losses in sprinting distance and maximum velocity during such scenarios. From a practical perspective, effective playing time and its general and position-specific effects should be taken into account when interpreting physical match performance of professional soccer players. Consequently, the information gained can facilitate load management practices and training design.
